# Uterine Artery Embolization on Serum β-HCG Levels, Fertility Function and Clinical Efficacy in Patients With Cesarean Uterine Scar Pregnancy

**DOI:** 10.3389/fsurg.2022.838879

**Published:** 2022-02-02

**Authors:** Wenyang Zhu, Xiaofang Zhang, Chang Liu, Yang Liu, Wei Xu

**Affiliations:** ^1^Department of Interventional Radiolody, The Affiliated Huaian Hospital of Xuzhou Medical University, Huaian, China; ^2^Department of Ultrasound, The Huaian Clinical College of Xuzhou Medical University, Huaian Maternity and Children Hospital, Huaian, China; ^3^Department of Gynaecology, The Affiliated Huaian Hospital of Xuzhou Medical University, Huaian, China; ^4^Department of Gynecology, The Huaian Clinical College of Xuzhou Medical University, Huaian Maternity and Children Hospital, Huaian, China; ^5^Department of Interventional Radiolody, The Affiliated Hospital of Xuzhou Medical University, Xuzhou, China

**Keywords:** cesarean section, uterine scar pregnancy, uterine artery embolization, serum β-HCG, fertility function

## Abstract

**Objective:**

To analyze the therapeutic effect of uterine artery embolisation (UAE) in patients with cesarean section pregnancy (CSP) delivered by cesarean section and the effect on serum human chorionic gonadotrophin (β-HCG) levels and reproductive function.

**Methods:**

In total 142 patients with CSP, The control group (*n* = 71) received Methotrexate (MTX) with ultrasound monitoring after admission and the research group (*n* = 71) was treated with UAE on basic of the control group. The two groups were compared in terms of treatment outcome, intraoperative bleeding, bed activity, vaginal bleeding and length of hospital stay, and serum follicle stimulating hormone (FSH), oestradiol (E2), luteinising hormone (LH) and β-HCG levels at 1 month postoperatively. The clinical symptoms (normalization of β-HCG and return of menstruation) and clinical outcomes (normal pregnancy, recurrent scar pregnancy) were compared between the two groups, as well as the occurrence of post-operative complications in both groups.

**Results:**

Compared with the control group, the research group had a higher overall near-term effective rate, a lower recurrence rate of CSP in pregnancy, and a lower complication rate (*P* < 0.05); meanwhile, the time to get out of bed, postoperative vaginal bleeding, length of hospital stay, normalization of serum β-HCG, and return to menstruation were shorter in the research group than in the control group (*P* < 0.05); In addition, serum FSH, E2, LH and β-HCG levels improved better in the research group compared with the control group 1 month after surgery (*P* < 0.05).

**Conclusion:**

The treatment of CSP patients with UAE can reduce the amount of intraoperative bleeding and the duration of vaginal bleeding, promote the improvement of patients' clinical symptoms, have less impact on the disruption of patients' sex hormone balance, reduce patients' surgical risks to a greater extent, preserve patients' normal fertility, and have better application.

## Introduction

Cesarean uterine scar pregnancy (CSP) is defined as a gestational sac lodging with the cesarean scar of the uterus. It is a relatively uncommon but high risk index ectopic pregnancy and is one of the distant complications of cesarean delivery (1). The exact etiology of the condition is not yet fully understood. Some studies (2, 3) have confirmed that in the period following cesarean section, the uterine scar becomes vascularized and rich in blood supply, which may explain the tendency of fertilized eggs to migrate to the vascularized area around the scar for implantation and implantation. It has also been found (4, 5) that more than 70% of CSP after cesarean section occur in those with a history of more than 2 cesarean sections, suggesting that enlarged, fibrotic, poorly formed local blood vessels and poor healing of the uterine scars after multiple cesarean sections are associated with the development of ectopic pregnancy there. In recent years, with the introduction of China's two- and three-child policy and the opening up of the public's ideology, the cesarean section rate of pregnant women has shown a clear upward trend, and the incidence of CSP has gradually increased. As a distant complication of cesarean delivery, CSP can lead to severe bleeding or even haemorrhagic shock in patients, with the possibility of uterine rupture and life-threatening effects of continued pregnancy (6–8).

Currently, there are many treatment options for CSP, including conservative treatment with methotrexate (MTX) alone and curettage, but the drawbacks of these options have gradually emerged with the widespread clinical application (9). The risk of hemorrhage and recurrence of CSP during re-pregnancy with medication alone is high, and is now often used as an adjunctive treatment during surgery; unclear indications for uterine removal can easily lead to intraoperative hemorrhage and even require uterine removal to preserve the patient's life and deprive the patient of fertility (10, 11). Uterine artery embolization (UAE) is a new minimally invasive interventional procedure that can rapidly and effectively control massive vaginal bleeding due to vascular injury. It has the advantage of being minimally invasive, with fewer side effects and fewer postoperative complications than uterine artery ligation, internal iliac artery ligation or hysterectomy, which were previously used to control massive vaginal bleeding (12, 13). In recent years this interventional technique has been widely used in the field of obstetrics and gynecology, especially in the treatment of postpartum hemorrhage (14), uterine fibroids (15) and cervical pregnancy (16), but there are still clinical concerns about whether UAE treatment of CSP will impair patients' reproductive function. In this trial, we treated CSP patients with UAE and analyzed its therapeutic effects as well as its impact on patients' serum Human chorionic gonadotropin (β-HCG) levels and fertility function.

## Materials and Methods

### Materials

#### Case Collection

A total of 142 patients with CSP requiring surgical treatment were selected and collected from May 2020 to May 2021 in our hospital. The control group received methotrexate (MTX) combined with B ultrasound-monitored clearance after admission, and the research group received MTX+UAE+B ultrasound-monitored clearance after admission. Baseline data on age, pregnancy, delivery, cesarean section, miscarriage, gestational week, gestational typing, gestational sac diameter and preoperative HCG level were collected from the two groups and the results showed no statistically significant differences (*P* > 0.05, Table 1) and were comparable.

**Table 1:** Comparison of baseline demographic information between the two groups of patients.

**Data**		**Control group (*n =* 71)**	**Research group (*n =* 71)**	**t/ χ^2^ value**	***P* value**
Age (years, Mean ± SD)		31.59 ± 5.26	32.41 ± 4.87	0.964	0.337
No. of pregnancies (times, Mean ± SD)		3.08 ± 1.45	3.00 ± 1.26	0.351	0.726
Number of deliveries (times, Mean ± SD)		1.25 ± 0.43	1.17 ± 0.38	1.175	0.242
Number of cesarean sections (times, Mean ± SD)		1.16 ± 0.34	1.17 ± 0.38	0.147	0.884
Number of abortions (times, Mean ± SD)		1.89 ± 1.36	1.92 ± 1.40	0.130	0.897
Pregnancy time (weeks, Mean ± SD)		8.78 ± 3.25	8.46 ± 2.96	0.613	0.541
Gestational sac diameter (cm, Mean ± SD)		4.57 ± 2.24	4.83 ± 2.08	0.717	0.485
Preoperative β-HCG (IU/L, Mean ± SD)		13,158.44 ± 1,302.59	13,136.47 ± 1,289.26	0.101	0.918
Exogenous (*n*, %)	Yes	42 (59.15)	46 (64.79)	0.478	0.489
	No	29 (40.85)	25 (35.21)		
Living embryo (*n*, %)	Yes	25 (35.21)	20 (28.17)	0.813	0.367
	No	46 (64.79)	51 (71.83)		

#### Diagnostic Criteria

1. Clinical symptoms: history of stop menstruation, positive urine pregnancy test with or without irregular vaginal bleeding and abdominal pain. 2. Ultrasonography was diagnosed (17) as follows: 1. No gestational sac was seen in the normal part of the uterine cavity and cervical canal; 2. Gestational sac or mass was visible in the isthmus incision; 3. Abundant blood flow was seen around the gestational sac and within the mass; 4. Lack of continuity of the myometrium in the cross-section through the gestational sac.

#### Inclusion Criteria

1. Meeting the above diagnostic criteria; 2. Patients had a clear previous history of cesarean section; 3. No relevant treatment prior to this admission; 4. No embryos in the uterine cavity by ultrasound; 5. Patients voluntarily signed an informed consent form with complete and uncompromised clinical information.

#### Exclusion Criteria

1. Patients with combined immune system disorders, infections, malignancies, hematological disorders; 2. Patients with ruptured gestational sacs; 3. Patients with severe coagulation abnormalities; 4. Patients with severe cardiac, hepatic, renal and other organ insufficiencies; 5. Patients with other combined pathologies in the uterine region; (6) combined cognitive impairment.

### Methods

#### Treatment Methods

After admission, all patients underwent routine blood routine, blood biochemistry, blood coagulation, electrocardiogram, and chest X-ray examinations, and there were no abnormalities.

Patients in the control group were treated with MTX+B ultrasound-monitored curettage: MTX was given as a single intravenous injection of l00 mg upon admission, and the patient's blood β-HCG level was monitored. When the patient's blood β-HCG decreased to 100 U/L, curettage was performed under B ultrasound-monitored. All operations were performed under transabdominal ultrasound guidance with lidocaine paracervical nerve block anesthesia and the use of an electric suction and scraping spoon to remove the pregnancy, being as careful as possible when scraping the pregnancy residue from the cesarean scar defect to avoid serious complications such as uterine rupture. If active bleeding was seen during clearance, the uterine cavity was filled with iodoform gauze and the gauze was removed after 24 to 48 h. If the above conservative treatment failed, transabdominal gestrectomy or hysterectomy was performed.

Patients in the research group were treated with MTX+ UAE+ B ultrasound-monitored for clearance: intraoperative disinfection of the cavity towel and Seldinger femoral artery puncture was performed. The operator searched for a femoral artery puncture site in the patient's right lower limb, followed the course of the femoral artery after routine anesthesia, inserted the needle, placed the cannula into the arterial sheath, withdrew the puncture needle and introduced the ultra-smooth guidewire. Then the arteriogram was performed to determine the status of the uterine artery blood supply according to the display. The embolization of the uterine artery was indicated by the slow injection of diluted MTX 100 mg before embolization, followed by embolization of the uterine artery with gelatin sponge pellets until the signal of blood flow in the uterine artery disappears. Curettage was performed within 48–72 h of UAE (The operation was the same as that of the control group).

Postoperatively, all patients were routinely given antibiotics to prevent infection and urethral tubes were left in place for 1~2 d. Patients' serum β-HCG levels and vaginal bleeding were closely monitored postoperatively.

#### Observation Indicators

The operation-related indexes such as operation time, intraoperative bleeding, time of getting out of bed, vaginal bleeding and hospital stay were recorded for both groups; the occurrence of postoperative complications such as fever, lower abdominal pain, vaginal bleeding and uterine adhesions were counted for both groups. Patients were advised to follow up regularly after discharge and were advised to have their serum β-HCG measured weekly as an outpatient until it dropped to a normal value. Serum follicle stimulating hormone (FSH), estradiol (E2), luteinizing hormone (LH) and β-HCG levels were measured preoperatively and at 1 month postoperatively. Ultrasound may be repeated once a month to monitor post-operative uterine recovery, and patients were followed up in clinic or by telephone for menstrual recovery, pregnancy and recurrence of CSP.

Recent efficacy assessment: Cured: blood β-HCG decreased to normal, vaginal bleeding stopped and abdominal pain disappeared; Effective: blood β-HCG decreased and was close to normal, vaginal bleeding decreased and ultrasound showed a smaller pelvic mass; Ineffective: blood β-HCG remained unchanged or even increased, ultrasound indicated that the pelvic mass remained unchanged or increased, or intra-abdominal bleeding occurred and required secondary surgical treatment. Total effective rate = (cured + effective) cases/total number of cases ×100%.

#### Statistical Methods

The study used SPSS 20.0 to manipulate all figures and Graghpad Prism 8 to create charts for statistics. The mean ± standard deviation (Mean ± SD) was used to represent the econometric information consistent with normal distribution, and the *t*-test was carried out; the caseload and composition ratio were used to represent the count information, and the χ^2^ test was carried out. *P* < 0.05 represented a statistically meaningful difference.

## Results

### Comparison of Recent Outcomes Between the Two Groups

**Figure 1:**
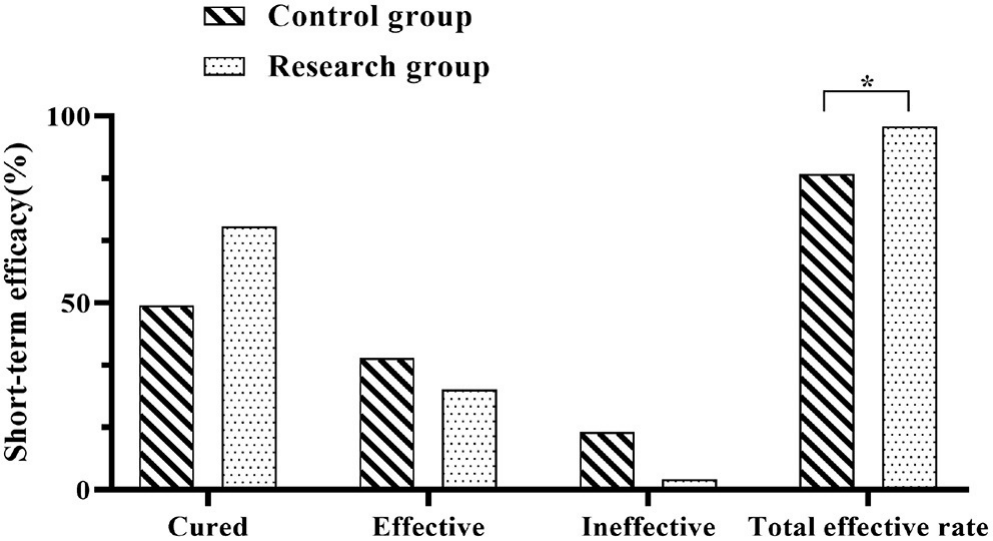
Comparison of recent outcomes between the two groups. Comparison between the two groups, *P < 0.05.

The recent outcomes of both groups were determined and the results were recorded. In the control group, 35 cases were cured, 25 cases were effective and 11 cases were ineffective, with a total effective rate of 84.61% (60/71). In the research group, there were 50 cured cases, 19 effective cases and 2 ineffective cases, with an overall effective rate of 97.18% (69/71). The recent efficacy rate showed significantly higher in the research group when compared against the control group (*P* < 0.05) (Figure 1).

### Comparison of Surgery-Related Indicators Between the Two Groups

**Figure 2:**
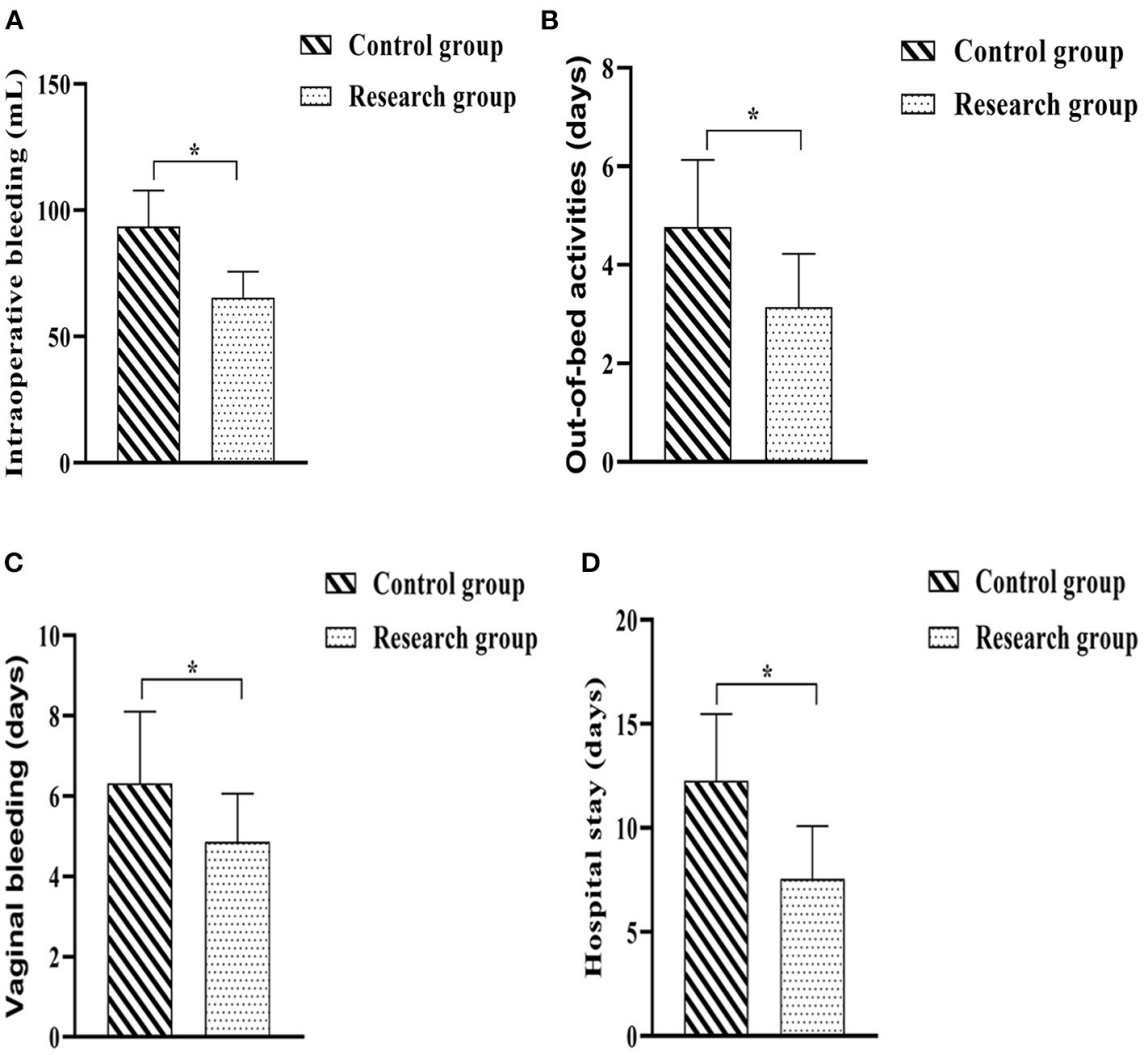
Comparison of surgery-related indicators between the two groups. **(A)** Intraoperative bleeding; **(B)** Out-of-bed activities; **(C)** Time of vaginal bleeding; **(D)** Hospital stay. Comparison between the two groups, *P < 0.05.

The operation-related indicators of both groups were recorded and statistically analyzed. The outcome revealed that the intraoperative bleeding was less in the research group than in the control group (*P* < 0.05); the postoperative time to bed, postoperative vaginal bleeding time and hospital stay in the research group when compared against the control group (*P* < 0.05) (Figure 2).

### Comparison of Reproductive Hormone Levels Between the Two Groups Before and 1 Month After Surgery

**Figure 3:**
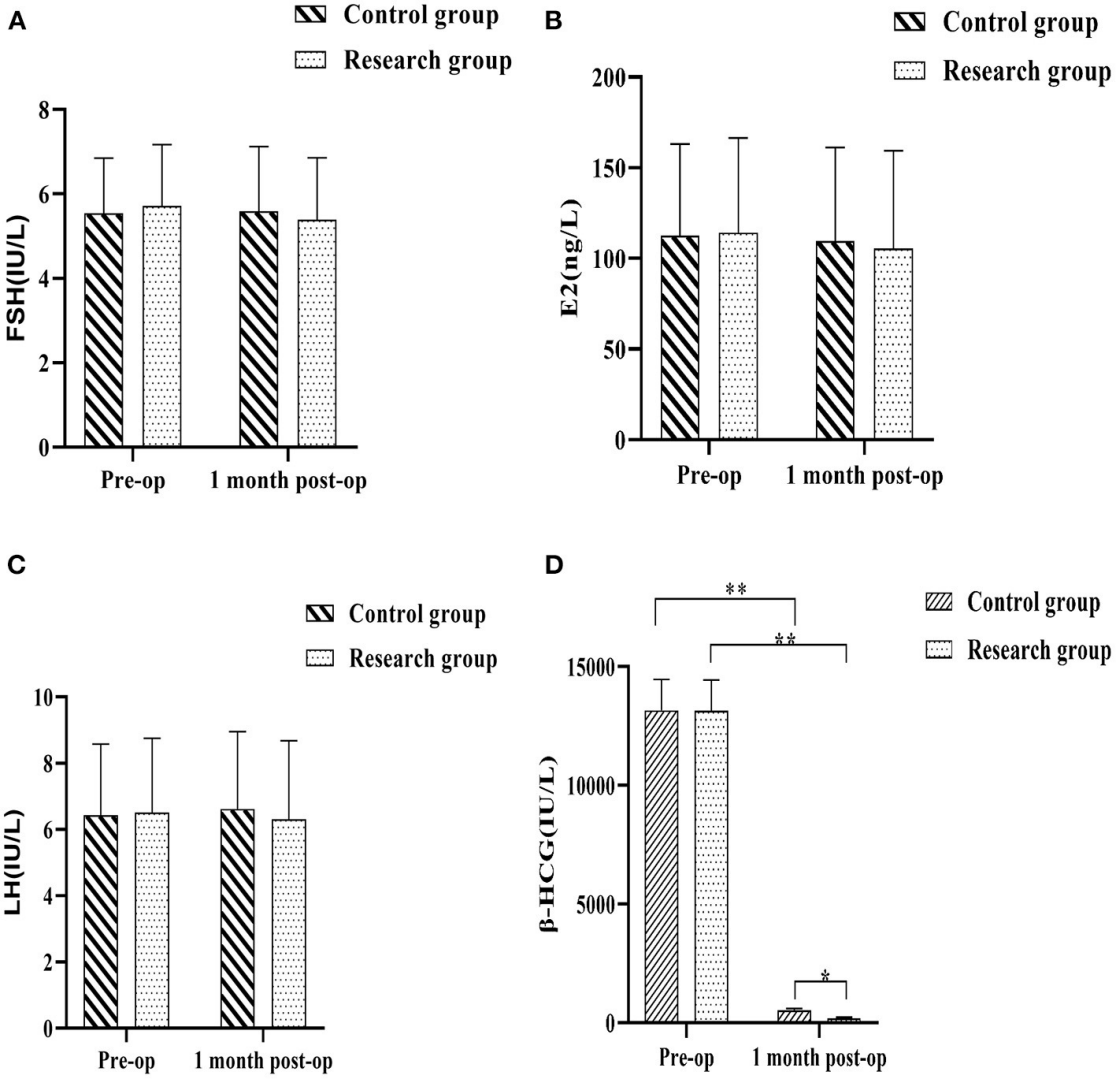
Comparison of reproductive hormone levels between the two groups before and 1 month after surgery. **(A)** follicle stimulating hormone (FSH); **(B)** estradiol (E2); **(C)** luteinizing hormone (LH); **(D)** Human chorionic gonadotropin (β-HCG). Comparison between the two groups over the same period, *P < 0.05; compared with the same group pre-op, **P < 0.05.

The blood samples of the patients were collected before operation and 1 month after operation to test the levels of reproductive hormones and recorded. The results showed that there would be no meaningful statutory variation in the pre-op serum FSH, E2, LH and β-HCG levels in either group (*P* > 0.05). The differences in serum FSH, E2 and LH levels between the control group and the research group at 1 month postoperatively were not statistically significant, and the contrast in plasma FSH, E2 and LH levels of each group at 1 month postoperatively and immediately prior to treatment were not considered of any statistical importance (*P* > 0.05). The serum β-HCG levels in the two groups decreased at 1 month after surgery compared with the preoperative levels, with the research group being lower when compared against the control group(*P* < 0.05) (Figure 3).

### Comparison of the Improvement of Clinical Symptoms and Pregnancy Outcome Between the Two Groups

**Figure 4:**
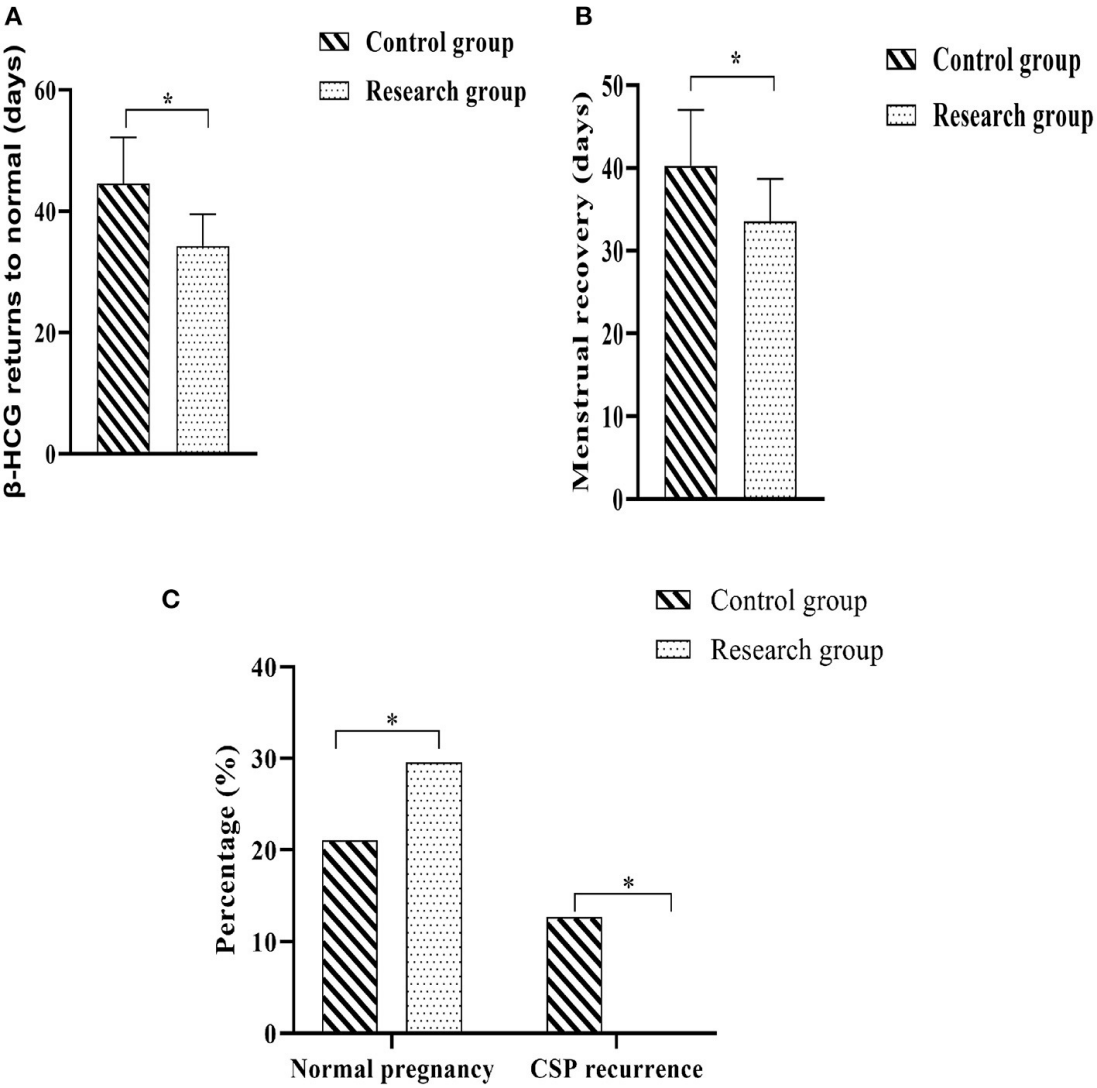
Comparison of the improvement of clinical symptoms and pregnancy outcome between the two groups. **(A)** Time to normalization of β-HCG; **(B)** Time to return to normal menstruation; **(C)** Normal pregnancy rate and CSP recurrence rate. Comparison between the two groups, *P < 0.05.

The two groups were followed up regularly, and the improvement of clinical symptoms and pregnancy outcomes during the follow-up period of the two groups were counted. The results showed that the time for serum β-HCG to return to normal and the time for menstruation to return to normal in the research group were shorter when compared against the control group (*P* < 0.05). There were 21 normal pregnancies (29.57%) in the research group and 15 normal pregnancies (21.13%) in the control group. A quick glance at the results of the regular pregnancies carried out among each group showed that there would be no statistically significant difference in the regular pregnancy rates in either group (*P* > 0.05). There were 0 cases (0.00%) of recurrent CSP in the research group and 9 cases (12.68%) of recurrent CSP in the control group, and a comparison of the recurrent CSP in the two groups showed that the rate of recurrent CSP in the research group was lower when compared against the control group (*P* < 0.05) (Figure 4).

### Comparison of Post-operative Complications Between the Two Groups

**Figure 5:**
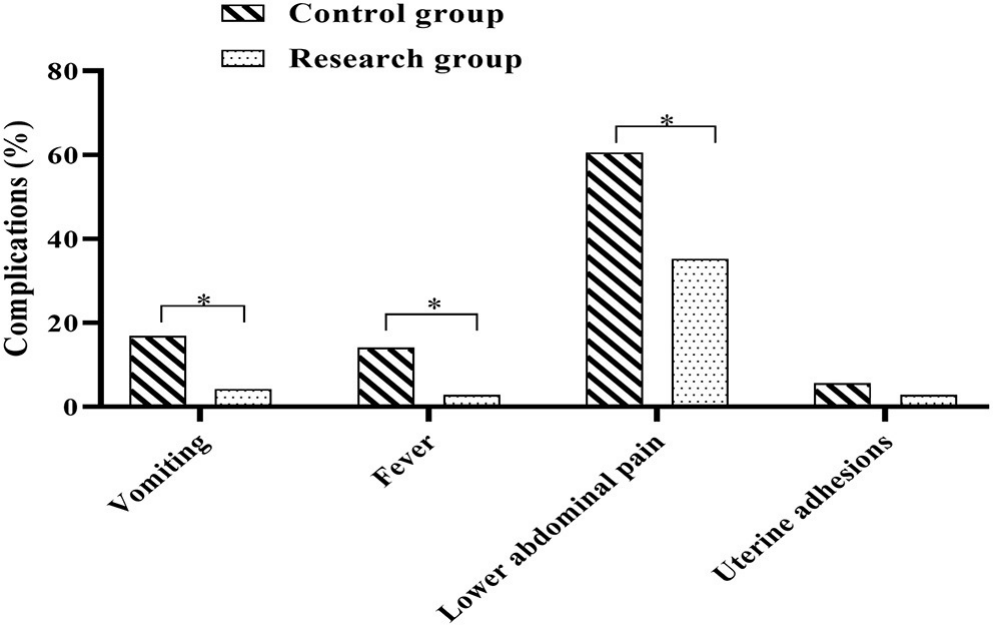
Comparison of post-operative complications between the two groups. Comparison between the two groups, *P < 0.05.

Postoperative complications were observed and recorded in both groups. In the control group, there were 12 cases of postoperative vomiting (16.90%), 10 cases of fever (14.08%), 43 cases of lower abdominal pain (60.56%) and 4 cases of uterine adhesions (5.63%); in the research group, there were 3 cases of postoperative vomiting (4.23%), 2 cases of fever (2.82%), 25 cases of lower abdominal pain (35.21%) and 2 cases of uterine adhesions (2.82%). Analysis of the complications in both groups showed that the incidence of vomiting, fever and lower abdominal pain in the research group was lower when compared against the control group (*P* < 0.05) (Figure 5).

## Discussion

As a long-term complication after cesarean section, CSP can lead to uterine rupture and placental implantation, increasing the incidence of hemorrhage and affecting sexual and reproductive function if not managed correctly (18, 19). The pathogenesis of CSP is not fully understood, but most scholars support the uterine incision defect theory (20, 21), which states that the uterine incision site is not fully healed after lower uterine cesarean section and is defective, thus making it easy for fertilized eggs to implant in the uterine incision scar where the endometrial defect exists. It has also been shown (22, 23) that the development of CSP may be closely related to the low position of the cesarean incision, multiple cesarean sections, defective suturing technique and postoperative incision healing, and wide scars. The currently accepted principles of CSP treatment (24) are early diagnosis, early termination of pregnancy and reduction of complications in order to preserve the patient's reproductive function reduce the trauma caused by surgery to the patient and achieve better outcomes.

In the past, hysterectomy was used as the only treatment option for CSP to avoid maternal mortality. In recent years, however, with the widespread use of ultrasound, CSP has been able to be diagnosed clearly at an early stage and conservative treatment has been widely used in clinical practice (25). Currently commonly used programs include systemic drug therapy, surgical therapy, and drug combined surgical therapy. MTX is a conservative drug commonly used in clinical practice, which can effectively preserve fertility by inhibiting trophoblast proliferation and causing necrosis of the villi cells, resulting in embryocidal effects (26, 27). UAE is an interventional procedure in which fresh gelatin sponge particles are introduced into the uterine artery to rapidly cause platelet coagulation and the formation of a thrombus, which can act as an embryocidal agent by blocking the blood supply to the embryo and can also greatly reduce the incidence of hemorrhage and preserve the patient's fertility (28, 29). In this study, the combination of local application of MTX, UAE and curettage for CSP patients showed that the intraoperative bleeding was lower in the research group than in the control group, and the time to get out of bed, postoperative vaginal bleeding, hospital stay, return to normal serum β-HCG and return to normal menstruation were all significantly shorter in the research group than in the control group; the recent efficacy of the research group was significantly higher than that of the control group (*P* < 0.05). It is possible that the thrombus has formed in the uterine artery and the ischaemic and hypoxic state has developed at the lesion by the time the uterine clearance is performed 48 to 72 h after UAE, which reduces the occurrence of intraoperative hemorrhage to a greater extent, decreases the amount of intraoperative bleeding and also shortens the duration of vaginal bleeding and hospital stay to a certain extent, which facilitates the patient's recovery (30). In this study, the levels of serum FSH, E2, LH and β-HCG and other reproductive hormones were also measured at different times, and the results showed that except for significant changes in serum β-HCG levels, the differences in the levels of other indicators between preoperative and 1 month postoperative were not significant. It is suggested that UAE treatment improves serum β-HCG levels in CSP patients without adversely affecting the secretory function of their ovaries. In addition, there was no difference in the normal pregnancy rate between the two groups during the follow-up period, but the recurrence rate of CSP in the research group was significantly lower than that in the control group, suggesting that UAE treatment is less damaging and more curative, and can preserve the patient's reproductive function to a greater extent, and has a good prognosis for pregnancy outcomes.

The results of this study also showed that the incidence of complications such as vomiting, fever and lower abdominal pain were significantly lower in the study group than in the control group. Analysis of the reasons for this: UAE interventions are able to form embolisms that block the circulation to the uterus, causing necrosis after the lesion is placed in an ischaemic and hypoxic environment (31). The local injection of MTX into the uterus prior to embolisation allows the local concentration of the drug to be at a high level, enabling the trophoblast to atrophy within a relatively short period of time. As the lesions become necrotic and the trophoblast cells are eliminated, the local concentration of MTX in the uterus is significantly reduced and less drug remains, thus reducing to a greater extent the risk of complications such as fever and vomiting associated with the application of MTX to the patient (32).

In conclusion, the use of UAE in CSP patients can reduce intraoperative bleeding and the duration of vaginal bleeding, promote the improvement of patients' clinical symptoms, have less impact on the disruption of patients' sex hormone balance, reduce patients' surgical risks to a greater extent, preserve patients' normal fertility, and have better results.

## Data Availability Statement

The original contributions presented in the study are included in the article/supplementary material, further inquiries can be directed to the corresponding author/s.

## Ethics Statement

The studies involving human participants were reviewed and approved by the Affiliated Huaian Hospital of Xuzhou Medical University. The patients/participants provided their written informed consent to participate in this study.

## Author Contributions

WZ and XZ are mainly responsible for data statistics and paper writing. CL and YL are mainly responsible for research design and result testing. WX is mainly responsible for the guidance of the entire research process. All authors contributed to the article and approved the submitted version.

## Conflict of Interest

The authors declare that the research was conducted in the absence of any commercial or financial relationships that could be construed as a potential conflict of interest.

## Publisher's Note

All claims expressed in this article are solely those of the authors and do not necessarily represent those of their affiliated organizations, or those of the publisher, the editors and the reviewers. Any product that may be evaluated in this article, or claim that may be made by its manufacturer, is not guaranteed or endorsed by the publisher.
